# Climate‐Driven Increase in Transmission of a Wildlife Malaria Parasite Over the Last Quarter Century

**DOI:** 10.1111/gcb.70550

**Published:** 2025-10-20

**Authors:** Angela Nicole Theodosopoulos, Fredrik Andreasson, Jane Jönsson, Johan Nilsson, Andreas Nord, Lars Råberg, Martin Stjernman, Ana Sofía Torres Lara, Jan‐Åke Nilsson, Olof Hellgren

**Affiliations:** ^1^ Department of Biology Lund University Lund Sweden; ^2^ Faculty 2 Biology/Chemistry University of Bremen Bremen Germany

**Keywords:** blue tit, climate change, *Haemoproteus*, *Leucocytozoon*, long‐term study, malaria parasite, *Plasmodium*, vector‐transmitted parasite

## Abstract

Climate warming is expected to influence the prevalence of vector‐transmitted parasites. Understanding the extent to which this is ongoing, or has already occurred, requires empirical data from populations monitored over long periods of time, but these studies are sparse. Further, vector‐disease research involving human health is often influenced by disease control efforts that supersede natural trends. By screening for malaria parasite infections in a wild population of blue tits ( *Cyanistes caeruleus* ) in Northern Europe, over a 26‐year period, we tested whether prevalence and transmission changes were climate‐driven. We found that all three malaria parasite genera occurring in blue tits (*Haemoproteus*, *Plasmodium*, and *Leucocytozoon*) have increased significantly in their prevalence and transmission over time. The most common parasite in the study, *Haemoproteus majoris*, increased in prevalence from 47% (1996) to 92% (2021), and this was a direct consequence of warmer temperatures elevating transmission. Climate window analyses revealed that elevated temperatures between May 9th and June 24th, a time period that overlaps with the host nestling period, were strongly positively correlated with *H. majoris* transmission in one‐year‐old birds. A warming climate during this narrow timeframe has had a demonstrable impact on parasite transmission, and this has favored an increase in the prevalence of parasites in wild birds in a temperate region of Europe. While more challenging to measure, similar implications of climate warming on human vector‐disease systems might be occurring. It is therefore critical that we understand what specific aspects of malaria parasite development and transmission are most influenced by climate warming, for the benefit of human and wildlife health.

## Graphical abstract

Climate warming is shaping ecosystems in several ways, and this includes changes in the dynamics of wildlife disease transmission. Over 26 years, avian malaria parasites have become much more common in a population of blue tits, small songbirds that we surveyed in southern Sweden. We found that warmer temperatures during the bird breeding season are tightly linked to this trend, and this may be due to climate‐induced impacts on the insects that transmit these parasites. Similar trends may be occurring in other regions, with potential consequences for both wildlife and human health.

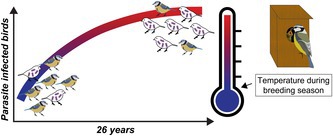

## 1 Introduction

A rise in vector‐borne diseases is a commonly expected effect that climate change can impose on ecosystems (Rocklöv and Dubrow 2020; Semenza and Suk 2018), but we lack long‐term empirical evidence that is necessary to support causation (Chala and Hamde 2021; Greening et al. 2025; Rocklöv and Dubrow 2020). For example, in 2010, an unprecedentedly warm year (Barriopedro et al. 2011), multiple Eurasian countries began experiencing outbreaks of West Nile Virus (primarily transmitted by *Culex* mosquitoes) (Paz et al. 2013). While this outbreak was linked to elevated summer temperatures, the lack of extensive long‐term data prevented pinpointing a direct causal effect from climate warming (Engler et al. 2013; Paz et al. 2013). The multifarious nature of climate change is at the helm of some vector population expansions (Cazelles et al. 2023; Duffy et al. 2024; Garamszegi 2011; Turner et al. 2020). However, connecting climate‐mediated vector trends with their associated host infection trends is challenging given the difficulty to disentangle the effects of climate from other factors that can influence transmission (Barrero Guevara et al. 2025; De Souza and Weaver 2024; Sadoine et al. 2024). Importantly, transmission is also affected by vector and host competence (Gervasi et al. 2015), habitat changes such as urbanization (Wilke et al. 2019), and vector control efforts (Bhatt et al. 2015).

Since 1897, malaria parasites have played a pivotal role in research on vector‐borne diseases. Mosquitoes were identified as the primary vehicle for their transmission to birds, and soon after, humans (Cox 2010). Broadly, malaria parasites are defined as the parasitic alveolates that comprise the order Haemosporida. These parasites are found in a diversity of vertebrate hosts, but the most common genera that infect birds are *Haemoproteus*, *Plasmodium*, and *Leucocytozoon*, each with their own set of vectors (biting midges, mosquitoes, and black flies, respectively; Valkiūnas 2004). As to humans, malaria, the disease that can result from haemosporidian parasite infections, can also be fatal to wildlife (LaPointe et al. 2012; Venkatesan 2024). A key concern amongst epidemiologists and conservationists is whether climate change will drive a global rise in malaria (Cazelles et al. 2023; Mordecai 2023; Sadoine et al. 2024), and addressing this concern requires an understanding of the mechanisms that shape the prevalence and transmission of the parasite. We have already seen severe consequences from malaria in endemic Hawaiian birds (LaPointe et al. 2012) and captive penguins (Hernandez‐Colina et al. 2021; Ings and Denk 2022), and a recent meta‐analysis revealed that avian malaria parasites negatively impact host fitness and phenology (Grabow et al. 2025). Warming temperatures may further facilitate range expansions of these parasites, such as their movement from lower to higher elevations (LaPointe et al. 2012; Zamora‐Vilchis et al. 2012), or allow malaria parasites currently restricted to the tropics to establish in temperate regions, facilitated by migratory birds (Fuller et al. 2012). Moreover, an additional concern is that such altitudinal or latitudinal range expansions will result in the accumulation of coinfections that can impose added costs on host survival (Marzal et al. 2008; Møller et al. 2013).

Human malaria was broadly eradicated in Europe in the middle of the 20th century (Boualam et al. 2021). Specifically, in Sweden, human malaria ( *P. vivax* ) was endemic in the 18th and 19th centuries, and historical data have shown that warmer summers were associated with outbreaks (Chen et al. 2021). Given the widespread control efforts targeting human malaria in more recent times (Andrews et al. 1950; Bhatt et al. 2015; Duffy et al. 2024), it is difficult to pinpoint the extent to which a rise in ambient temperatures influences parasite transmission, or how significant the effects of ongoing climate changes are on the systems if left unchecked. As control efforts are lacking in even the most well‐studied wildlife malaria systems (LaPointe et al. 2012), this enables their use for understanding how climate warming is shaping disease transmission. Importantly, only a few studies have investigated wildlife malaria in birds using data that span more than a decade of collection, despite the significance of this knowledge gap for both human health and wildlife conservation at a global level (Bensch et al. 2007; Otero et al. 2019; Wilkinson et al. 2016). We are therefore severely limited in our understanding of how these parasites might have already increased in prevalence given ongoing climate change. In Northern Hemisphere temperate zones, we can expect breeding seasons for many vectors and birds to coincide during temporal windows governed by climatic conditions. As such, long‐term studies targeting avian breeding seasons in these regions, combining host–parasite infection data with climate and vector data, are especially suitable for understanding how vector‐transmitted parasites are impacted by climate change.

**FIGURE 1:**
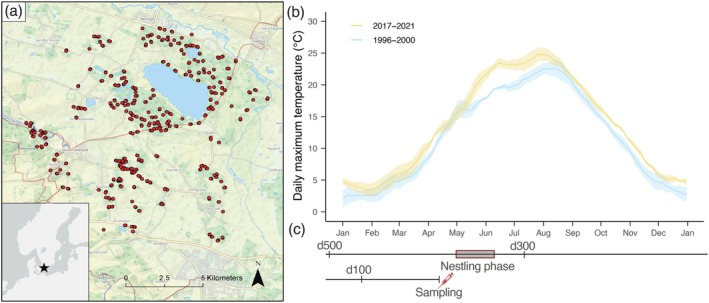
Long‐term survey of blue tits and climate trends at the Revinge field site. Between 1996 and 2021 blue tits were surveyed for malaria parasites at the Revinge field site in Southern Sweden at breeding locations indicated by red circles (a). 28 day rolling mean of the average maximum daily temperature for early and later years of the study. Ribbons represent ± one standard error (b). Birds were sampled during the nestling phase of their breeding season, and climate window analyses were allowed to extend 509 days prior to their sampling (nine days prior to d500, c).

We sought to address the above knowledge gap by harnessing a long‐term study of blue tits ( *Cyanistes caeruleus* ) in southern Sweden (Figure 1a). The blue tit is a small, typically non‐migratory passerine bird that is common in the Western Palearctic (Stenning 2018). Blue tits nest in cavities, and as such, nest boxes can be deployed to study their breeding (Stenning 2018). Since 1983, a population of blue tits has been studied in this way within the Revinge area, a study area approximately 20 km east of Lund (Andreasson et al. 2023). Along with their breeding data, blood samples have been collected from birds every year since 1996. We used blood samples collected from three five‐year periods, beginning in 1996 and ending in 2021, to identify malaria parasite infections. Based on data from the Swedish Meteorological and Hydrological Institute (SMHI 2024), the temperature in the region has become significantly warmer since the mid‐1990s (Figure 1b). Climate change has been hypothesized to facilitate a rise in many diseases resulting from vector‐transmitted parasites, in part due to vectors being ectotherms that persist better in warmer conditions (Rocklöv and Dubrow 2020). Temperature is also known to play a critical role in malaria parasite development. For example, both *P. vivax* and *P. falciparum* are known to require minimum temperatures of 16°C and 21°C, respectively, to complete their sporogonic cycle in mosquito vectors (Habtamu et al. 2022). In this study, we examined the relationship between temperature and malaria parasite prevalence in a wild bird population over a 26‐year study period to understand the implications of climate change on vector‐mediated parasite transmission in wildlife.

## 2 Materials and Methods

### 2.1 Hosts

**FIGURE 2:**
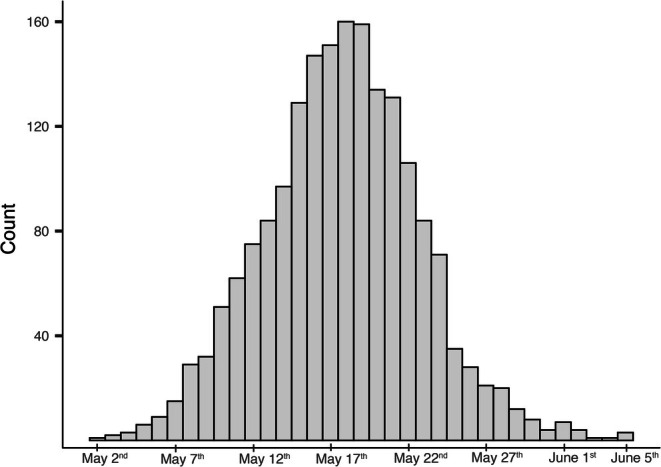
Blue tit breeding phenology. Histogram of hatching dates for all clutches hatched (*n* = 1882 clutches) spanning the three time periods of the study. Hatching took place between May 2nd and June 5th. Nestlings stay in the nest for ~20 days post‐hatching. Thus, the time window from hatching to fledging is from May 2nd to June 25th, an almost perfect overlap with the predicted climate window of May 9th to June 24th for *Haemoproteus* transmission (Figure 4).

At the Revinge field site, blue tits ( *Cyanistes caeruleus* ) have been monitored since 1983 using a network of around 450 nest boxes (centered on 55.69° N and 13.46° E and approximately 30 m in elevation) to study their breeding (Andreasson et al. 2023). Between the years 1996 and 2021, blood samples were collected from breeding adults between the months of April and July. All sampling was approved by the Malmö/Lund Animal Ethics Committee (permit nos. M 126–00; M 94–07; M 67–09; M 67–16; and 04705/2018). We extracted DNA from *n =* 1965 samples collected over 26 years and analyzed malaria prevalence and lineages across 15 breeding seasons in three five‐year time periods: early (1996–2000, *n* = 472), middle (2007–2011, *n* = 893), and later years (2017–2021, *n* = 600). Of all sampling events, 204 were from birds sampled more than once between field seasons. The dataset and code are available on Dryad (Theodosopoulos et al. 2025). Throughout the duration of the study, hatching dates occurred between the 2nd of May and the 5th of June (Figure 2). Given that tit nestlings on average stay in the nest for 20 days (Nilsson and Svensson 1996), the last fledglings will leave their nest on June 25th. Birds were aged and sexed based on plumage characteristics and the presence or absence of a brood patch (Svensson 1992).

### 2.2 Vectors

During the 2008 and 2009 field seasons, biting midges (vectors for *Haemoproteus* parasites; Valkiūnas 2004) were surveilled from nest boxes at the Revinge field site (Nilsson and Stjernman 2025). To identify individuals to species, DNA was extracted from 30 individuals, all morphologically identified as biting midges, and molecular identification was done by sequencing the mitochondrial *cox1* gene using two different combinations of primers (Videvall et al. 2013).

### 2.3 Parasites

#### 2.3.1 Multiplex PCR Screening

To simultaneously detect infections with *Haemoproteus*, *Plasmodium*, and *Leucocytozoon*, we used multiplex PCR methods (Ciloglu et al. 2019). All birds were screened twice to increase the detection probability of infections with low parasitemia (see Supporting Information: Extended Methods). We used all samples from the study to assess prevalence (proportion of individuals infected) because this best reflects the overall parasite abundance in the total population of blue tits in a given year, and in turn, the yearly parasite population. As such, including all annual samples enabled us to detect the overall accumulation or loss of parasites in the host population.

#### 2.3.2 Parasite Lineage Identification

Variation in the mitochondrial *cytb* gene is commonly used to type genetic lineages of malaria parasites (Bensch et al. 2009). We typed genetic lineages using nested PCR, a commonly used practice, with previously described primers (Hellgren et al. 2004). PCRs were run separately to amplify (1) *Leucocytozoon* and (2) *Haemoproteus* and *Plasmodium*. For all nested PCRs, we included both negative controls and positive controls for each of the three malaria parasite genera.

Using samples that tested positive from multiplex PCR, nested PCRs were conducted on a subset of 656 samples for *Haemoproteus* and *Plasmodium*, and a subset of 83 *Leucocytozoon* samples. *Plasmodium* infections were challenging to type given that many were co‐amplified with *Haemoproteus*, which is usually present with a higher parasitemia in the blood (Waldenström et al. 2004). As such, we were only able to type 94 *Plasmodium* samples.

Following amplification of PCR products, samples were sequenced using an ABI 3100 Sanger Sequencer (see Supporting Information: Extended Methods). We downloaded the resulting chromatogram files and uploaded them into Geneious Prime software (v2025.0.3; Kearse et al. 2012). Finalized sequences were aligned to available typed lineage references from the MalAvi database using the BLAST algorithm (Bensch et al. 2009; Johnson et al. 2008). To be assigned to a specific lineage, the sequences needed to have a 100% match with previously described lineages from the MalAvi database. Some sequences could not be typed to lineage due to the presence of multiple mixed peaks observed in chromatograms; this was most common for *Leucocytozoon*.

Molecular screening data and microscopical examination of blood smears confirmed that low prevalence in early years was not due to sample degradation. As expected, PCR data nearly always presented almost equal or higher prevalence estimates than blood smears (Table S1), in contrast to what would be expected if DNA samples had degraded and failed to amplify (Stjernman 2004).

#### 2.3.3 Modeling Prevalence and Transmission Over Time

To examine prevalence, we used all *n* = 1965 samples from the blue tit population. We additionally tested for transmission, but understanding temporal transmission trends requires hosts to have only experienced a single season of parasite exposure upon sampling. This is because birds often harbor low‐intensity chronic malaria parasite infections that can last multiple infection seasons (Knowles et al. 2010). We therefore used a subset of *n* = 1154 one‐year‐old birds to evaluate transmission. Thus, these individuals had only lived through one complete transmission year.

We used R (version 4.2.2) base functions to obtain the mean prevalence (proportion of individuals infected) and to estimate prevalence, as an effect of year, with a generalized linear model (GLM), using a binomial error distribution and a logit link function (R Core Team 2013). The model is as follows: logit (Prevalence of each parasite genus) = α + β(Year), where alpha is the intercept, and beta is the slope of change that corresponds with sampling year. We ran models separately for each malaria parasite genus. To estimate transmission, we applied the same model but only included one‐year‐old birds from the study. We used the R packages *visreg* (Breheny and Burchett 2017) and *ggplot2* (Wickham 2016) to visualize models overlayed with yearly mean prevalence values.

### 2.4 Modeling the Impact of Temperature on Parasite Transmission

#### 2.4.1 Climate Window Analyses

We conducted climate window analyses to detect potential climate signals for transmission of each of the three malaria genera (R package *climwin*; Bailey and Van De Pol 2016; Van De Pol et al. 2016). The analyses compare the association between potentially important environmental variables in all possible time windows within a selected timeframe and the response variable (i.e., parasite transmission) using AICc (Akaike Information Criterion with small sample size correction; Burnham and Anderson 2004), with ∆AICc being the difference between the candidate climate model and the baseline model (containing no climate data and only an intercept). In our case, we selected temperature as the environmental variable given that ectothermic vectors rely heavily on their surrounding ambient temperature (Rocklöv and Dubrow 2020).

We used daily mean, minimum, and maximum temperatures recorded by a weather station (Swedish Meteorological and Hydrological Institute, station Lund 53,430) approximately 20 km from the study site as environmental predictors (i.e., climate signals) in a GLM with yearly mean parasite transmission for all one‐year‐old birds as the response variable. Each of the three parasite genera was modeled separately, using a binomial error distribution and a logit link function (the same model formulation as for prevalence and transmission over time, but with the different climate variables added as covariates instead of the sampling year). Additionally, the total number of sampled individuals each year was added as a weighting factor. We allowed for both a linear and quadratic relationship between the environmental predictors and the response variable.

Birds were sampled during, or in conjunction with their breeding season in the spring (yearly median sampling date was between May 15th and June 7th), and malaria parasites are not detectable until approximately 14 days after infection (Valkiūnas 2004). Therefore, we set the upper limit for the time‐window to 14 days before the median date of the year with the latest median sampling date to reduce the possibility of detecting biologically irrelevant climate windows. Both biting midges (*Culicoides* spp.) and, to a lesser extent, black flies (Simuliidae) are known to be present in nest‐boxes during the nestling phase (Martínez‐de La Puente et al. 2009). Therefore, we extended the timeframe of possible climate windows back to January 1st the preceding year, to include potential climate signals during the period before hatching and the nestling phase of sampled birds. Thus, we evaluated all climate windows from May 24th and extending back to January 1st the preceding year. We also chose to exclude windows that were 10 days or shorter, as we considered such windows to be biologically implausible and thereby increasing the risk for false positives if included.

#### 2.4.2 Climate Window Fits

Testing all possible time‐windows within such a long timeframe (approximately 130,000 windows were tested going back to January 1st of the preceding year) increases the probability of finding a false positive, i.e., a signal that is not a true biological signal but merely due to chance. We therefore ran 1000 randomizations for each parasite‐signal combination, where the randomizations reshuffled the climate data. As such, within the dataset, any given date is paired with a randomly chosen climate value, thereby removing any climate signal from the data, and this generates ∆AICc‐values that can be compared to the ∆AICc‐value of the best performing model. We used these comparisons to estimate the possibility that the climate window found is a false positive, and we ran these randomizations for climate signals that were within two ∆AICc of the best performing model (Burnham and Anderson 2004). We were interested in a stable estimate of the best performing climate signal and its corresponding time‐window. Therefore, we consistently used the median opening and closing dates of all windows that together made up 95% of the model weights, i.e., the 95% model confidence set, instead of the single best performing window (Table S2).

#### 2.4.3 Path Analysis

To tease apart the effect of temperature from any year effects that are unrelated to temperature, we conducted a path analysis using the R package *piecewiseSEM* (Lefcheck 2016). The path analysis converts the paths between variables in a path diagram to a set of linear equations that can be analyzed independently (Grace 2006). The three paths estimated were (1) the effect of year on the temperature variable identified by *climwin*, (2) the effect of temperature on parasite prevalence, and (3) the direct effect of year on parasite prevalence. Transmission was modelled using a GLM with climate window temperature and year as continuous predictors, and yearly mean transmission as the response variable, using a binomial error distribution and a logit link function with the total number of sampled individuals each year added as a weighting factor. The effect of year on climate window temperature was modelled by linear regression with a Gaussian error distribution. Coefficients were standardized using a latent‐theoretical approach, as applied in *piecewiseSEM* (Grace et al. 2018), allowing for a direct comparison of relative importance between pathways. By uncoupling the effect of year from temperature, we were able to quantify how climate influenced parasite transmission. Since we were focused on the relationship between temperature and transmission, we only used data from one‐year‐old birds for all climate window and subsequent structural equation modeling analyses.

## 3 Results

### 3.1 Lineages and Prevalence Changes

All typed *Haemoproteus* samples were of the same genetic lineage (PARUS1) belonging to the morpho‐species *Haemoproteus majoris* (Nilsson et al. 2016). The majority of identified *Plasmodium* lineages were either TURDUS1 (52%) or BT7 (38%), both of which belong to the same species, *Plasmodium circumflexum* (Valkiūnas et al. 2022). *Leucocytozoon* was the most diverse genus with at least eight different genetic lineages, and not all infections could be identified to lineage from molecular methods due to multi‐lineage coinfections (Table S3).

*Haemoproteus* experienced a significant prevalence increase over the study period (*p* < 2.0 × 10^−16^) where prevalence rose from 47% (1996) to 92% (2021, Table S4). *Plasmodium* and *Leucocytozoon* showed more prevalence fluctuations during early and middle sampling years, but a consistently higher prevalence during later years (Table S4). As such, infections with *Plasmodium* and *Leucocytozoon* were significantly more common in later years (*p* = 4.5 × 10^−16^ and *p* = 2.3 × 10^−6^, respectively).

### 3.2 Transmission Changes

**FIGURE 3:**
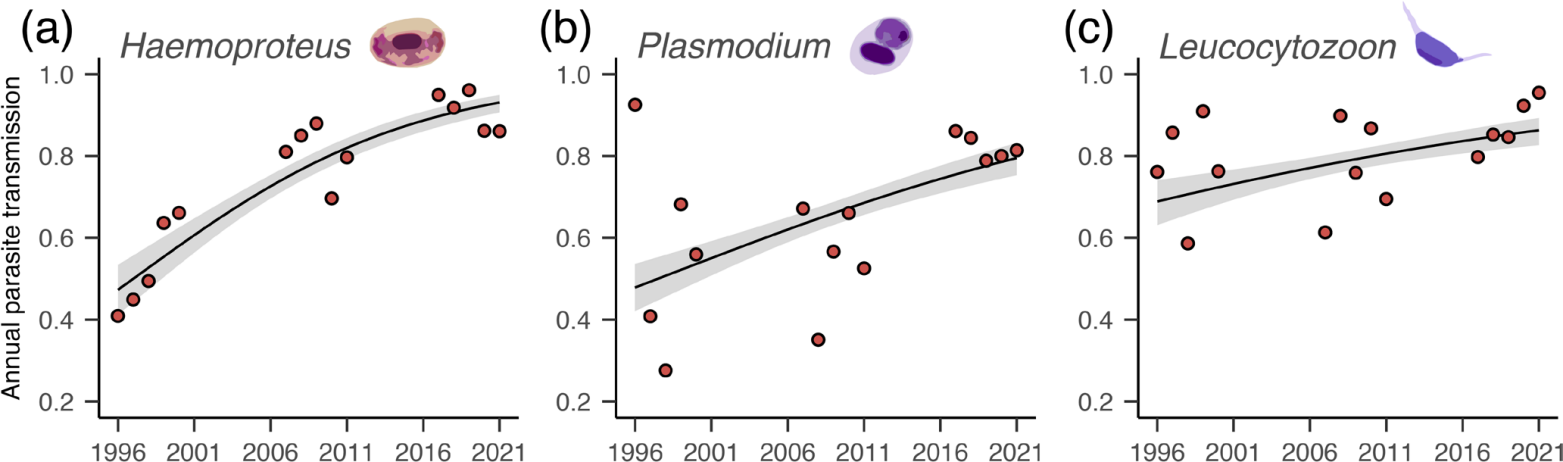
Transmission trends. Estimated annual transmission in relation to year for *Haemoproteus* (a), *Plasmodium* (b), and *Leucocytozoon* (c) in one‐year‐old birds with 95% confidence intervals shown. Points show the mean prevalence for each year.

For *Haemoproteus*, the temporal increase in transmission was equivalently significant for one‐year‐old birds, as the prevalence increase for the broader population (*p* < 2.0 × 10^−16^, Figure 3a). *Plasmodium* transmission also increased significantly during the study period (*p* = 1.7 × 10^−12^, Figure 3b), as did *Leucocytozoon* (*p* = 6.0 × 10^−6^, Figure 3c).

### 3.3 Relationship Between Transmission and Temperature

**FIGURE 4:**
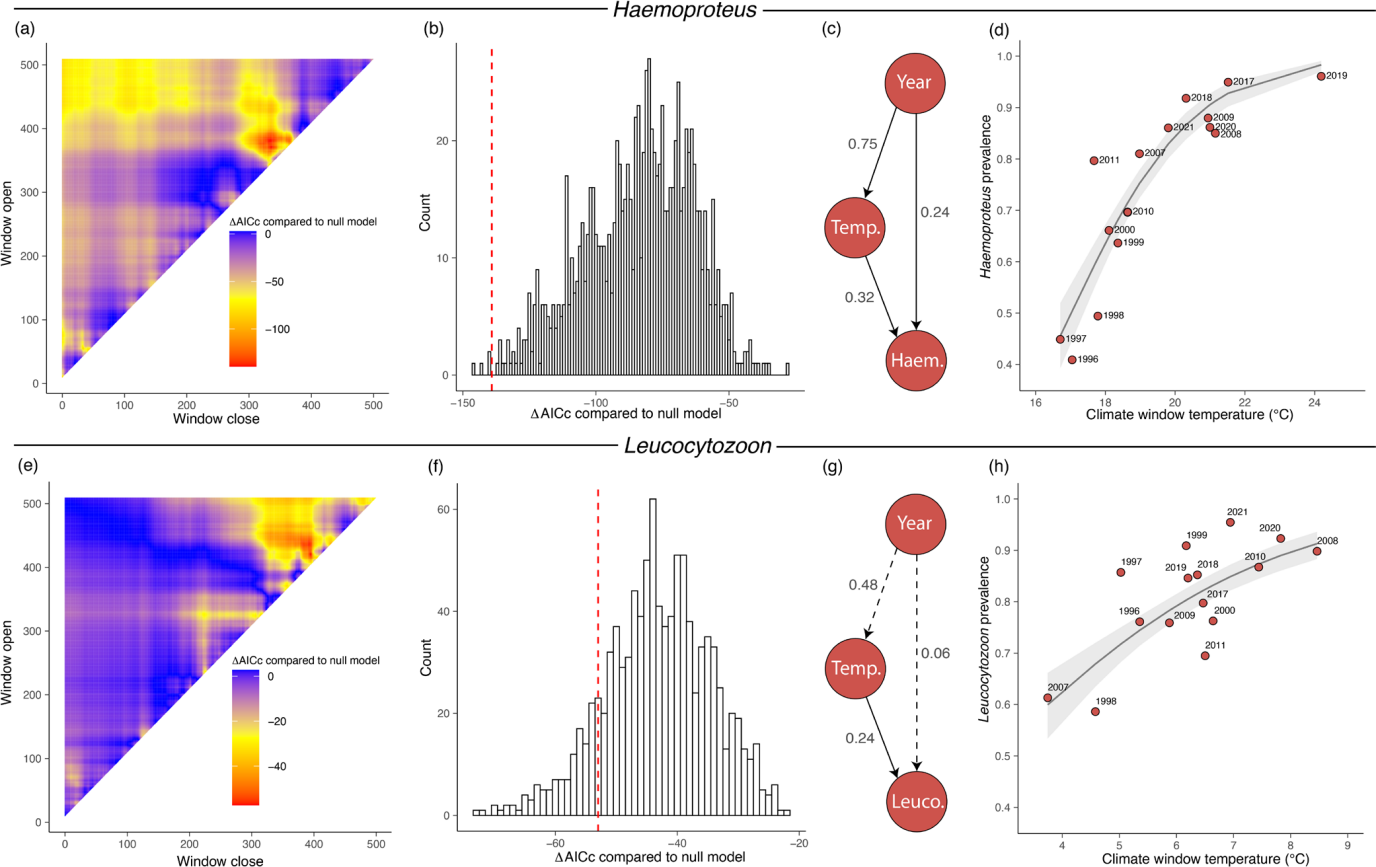
Climate windows and path analyses. ΔAICc from *Haemoproteus climwin* analysis for the selected climate signal model compared to the null model (containing no climate data but only an intercept) across all possible windows with the most likely window opening on May 9th (380 days before the reference day) and closing June 24th (334 days before the reference day), i.e., where the area with the lowest value for ΔAICc is located (red; a). ΔAICc of 1000 model runs on randomized data (histogram) compared to the obtained ΔAICc from the selected climate signal model, shown as dashed red line (b). Path model with standardized coefficients that reflect the relative importance of each path (c). Yearly *Haemoproteus* prevalence in one‐year‐old birds as a function of climate window temperature (d). Dots are raw values, and the smoothed line indicates predicted probabilities together with their 95% confidence intervals. For (e–h) the same outputs are shown for *Leucocytozoon* but with the most likely climate window opening on March 12th and closing on May 1st. Dashed lines in (g) indicate non‐significant (*p* > 0.05) effects.

**FIGURE 5:**
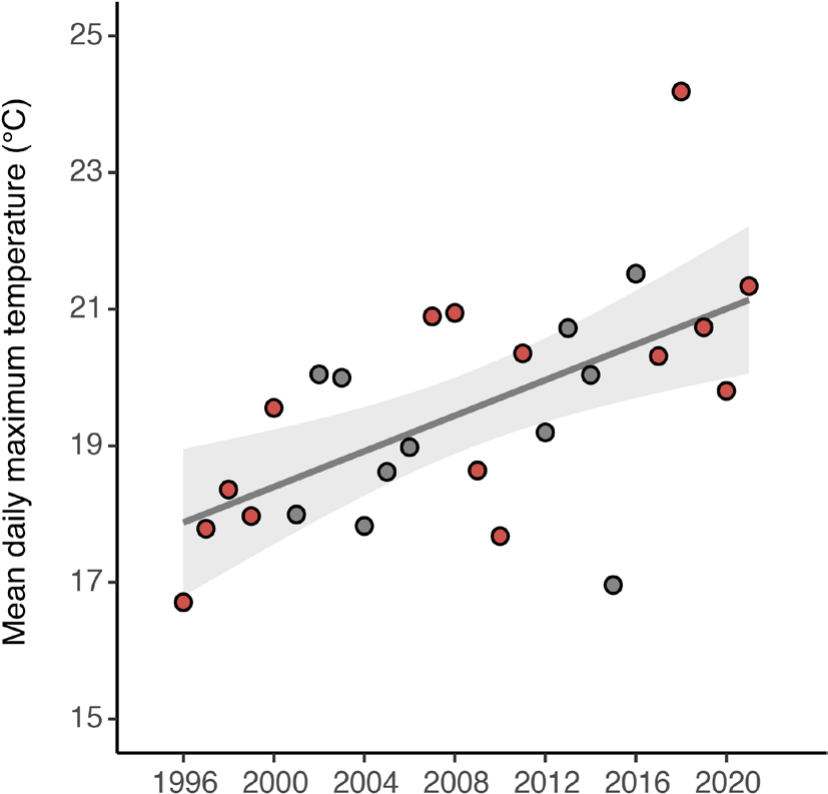
Climate window temperature for *Haemoproteus*. Mean daily maximum temperature and 95% confidence intervals during the climate window (May 9th – June 24th) for *Haemoproteus* (temperature = −242.4 + 0.13 × year, *p* = 1.3 × 10^−3^). Red dots indicate years with parasite sampling data.

*Haemoproteus* transmission had the highest supporting climate signal of all three genera (99.6% support, Figure 4a,b), based on the average daily maximum temperature in a window spanning May 9th to June 24th of the year before sampling. Notably, the climate window overlaps with the nestling period for hosts (May 2nd to June 25th) in the study system almost perfectly (Figures 1c and 2). The average daily maximum temperature within this climate window has notably risen over the whole 26‐year study period (Figure 5). Climate window temperature increased (± SE) with 0.17°C (± 0.04) for each year (t_13_ = 4.1, *p* = 1.2 × 10^−3^) and both temperature (z_12_ = 4.2, *p* = 2.6 × 10^−5^) and year (z_12_ = 3.7, *p* = 2.2 × 10^−4^) had a direct positive effect on parasite transmission (Figure 4c). The odds of getting infected increased with 39.8% for every °C increase in temperature (log odds ± SE: 0.33 ± 0.08, Figure 4d) and the odds of getting infected also increased with 5.8% each year (log odds ± SE: 0.06 ± 0.02, i.e., independently of temperature). The direct effect of temperature was approximately 33% stronger compared to the direct effect of year (standardized coefficients of 0.32 vs. 0.24, Figure 4c,d). Thus, a higher maximum daily temperature during the nestling stage correlated with more *Haemoproteus* transmission.

For *Leucocytozoon* transmission, the best window had 86.2% support for being a true signal and was based on the average daily mean temperature with a window spanning March 12th to May 1st during the previous year (Figure 4e,f). Climate window temperature had a direct effect on parasite transmission (z_12_ = 5.9, *p* = 3.2 × 10^−9^) where the odds of getting infected increased by 45.8% for every°C increase in temperature (log odds ± SE: 0.38 ± 0.06, Figure 4g). During this window, the mean daily temperature tended to increase with year (0.07°C ± 0.03°C; t_13_ = 2.0, *p* = 0.07) but there was no direct effect of year on parasite transmission (z_12_ = 1.2, *p* = 0.23). *Leucocytozoon* had notably low prevalence in 1998 and 2007 (Figure 4h) and both these breeding seasons were preceded by relatively cold years, when transmission would have taken place. The best supported climate window for *Plasmodium* had a low probability of being a true signal (73.1%, Table S2) and was therefore not analyzed further.

### 3.4 *Haemoproteus*‐Transmitting Vectors

Climate window analyses indicated that *Haemoproteus* transmission occurs when blue tits are nestlings; therefore, exposure to *Haemoproteus*‐transmitting vectors must be taking place during this time. We collected 30 samples of morphologically identified biting midges from nest boxes, and 17 had successful amplification of the mitochondrial *cox1* gene. These individuals were identified as *Culicoides pictipennis*, *C. kibunensis* , and *C. segnis* by aligning the resulting Sanger sequencing data to the NCBI database using the BLAST algorithm (Johnson et al. 2008). All three of these biting midge species are presumed vectors for *H. majoris* (Chagas et al. 2024).

**FIGURE 6:**
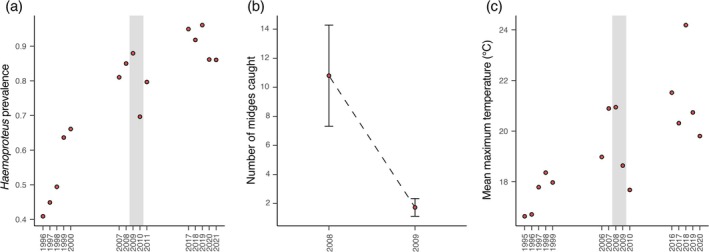
A link between *Haemoproteus* prevalence, vector abundance, and temperature variation. The average *Haemoproteus* prevalence for one‐year‐old birds (i.e., transmission) spanning each year of sampling shows notably higher prevalence in the 2009 vs. 2010 breeding season (a). The number of *Haemoproteus*‐transmitting biting midges recovered from nest boxes during a 4‐day trapping session was higher in 2008 compared with 2009 (Nilsson and Stjernman 2025), and this could potentially mean that more midges during the nestling period result in higher *Haemoproteus* prevalence the following year (b). Incidentally, the 2008 breeding season was warmer than in 2009, and this supports our expectation that there are more *Haemoproteus‐*harboring biting midges in warmer years than in cooler years (c).

Differences in biting midge abundance can vary annually, and this likely influences *Haemoproteus* prevalence in one‐year‐old birds the following year. We see some evidence to support this in a notable drop in *Haemoproteus* prevalence between 2009 and 2010 (Fig. 6a) corresponding to the reported lower biting midge abundance in 2009 compared to 2008 (Nilsson and Stjernman 2025; Fig. 6b). Additionally, based on averaged maximum temperatures within the climate window, 2008 was a warmer year than 2009 (Fig. 6c), consistent with a link between ambient temperature, the vector, transmission, and *Haemoproteus* prevalence. However, more years of data collection will be needed for this to be established as a general pattern.

## 4 Discussion

Despite the ubiquity of parasites, our understanding of their infection trends rarely extends beyond a snapshot in time (Hayward et al. 2022), limiting our ability to predict how their prevalence and transmission will change as the result of ongoing global climate warming (Rizzoli et al. 2019; Rocklöv and Dubrow 2020). By using a long‐term dataset spanning over a quarter century, our study is the first to unambiguously show a link between ambient temperature during a specific time window and the prevalence of a vector‐transmitted parasite. Specifically, over the past 26 years, the prevalence of *Haemoproteus majoris* (lineage PARUS1) has increased from moderate to high levels through an increase in annual transmission that likely takes place when birds are nestlings (May 9th—June 24th). More one‐year‐old birds are getting infections in recent times, and this transmission increase permeates to a rise in prevalence for the total population of hosts. Variation in biting midge abundance and host‐vector encounter rates may contribute to annual levels of PARUS1 transmission. Warmer temperatures can influence this dynamic by enhancing vector reproduction, development, and host‐seeking behavior, as observed in *Aedes* mosquitoes (Reinhold et al. 2018). Additionally, temperature limits are also important for *H. majoris* sporogonic development (Žiegytė et al. 2017) and gametogenesis (Valkiūnas 2004). Experiments that further explore host‐seeking behavior by vectors, the interplay between temperature and vector abundance, and the climatic conditions required for parasite development and transmission will be fruitful and can now be more targeted towards the relevant climate window and associated temperatures.

There are alternative explanations for the increase in PARUS1 prevalence in the blue tit population, but we consider them less likely. PARUS1 is a generalist parasite that infects a broad range of Passeriformes (Nilsson et al. 2016), making it unlikely that host morphology changes would result in the prevalence rise. A micro‐evolutionary increase in host susceptibility is another possibility, but we consider this to be outweighed by the observed link between temperature and vector abundance that contrasts with this explanation. It is also evident that PARUS1 is locally transmitted in Sweden, as it is found in juvenile birds before migration, and in resident bird species (Nilsson et al. 2016), suggesting that prevalence changes would not be influenced by introductions of the parasite from migratory birds. This is further supported by evidence that transmission shifts between tropical and temperate regions are rare over ecological time (Hellgren et al. 2007). Collectively, we consider changes in host‐vector dynamics and parasite development, resulting from climate warming, to be the most plausible drivers of the PARUS1 prevalence increase.

Compared to *Haemoproteus*, the predicted climate window for *Leucocytozoon* lies earlier in the season (from March 12th to May 1st, before eggs have hatched). The black flies (Family: Simuliidae) that transmit *Leucocytozoon* typically breed in flowing water (Adler and McCreadie 2019), and the water temperature of flowing streams at the Revinge field site might therefore play a role in determining when black flies emerge. An earlier vector emergence could mean more opportunities for *Leucocytozoon* transmission. *Leucocytozoon* is by far the most diverse of the malaria parasites that we sampled (at least eight different lineages). As such, there could be multiple black fly species transmitting these parasites, thus making it harder to accurately predict how temperature is linked to vector ecology and *Leucocytozoon* transmission.

For avian *Plasmodium* parasites, mosquitoes of the genera *Culex*, *Culiseta*, *Aedes*, and *Anopheles* are all described vectors (LaPointe et al. 2012), but the specific species that transmit *Plasmodium circumflexum* (lineages TURDUS1 and BT7) are not currently known (Valkiūnas et al. 2022). We suspect that these vectors are actively transmitting infections during a relatively broad span of the blue tit annual cycle, given our inability to identify any specific associated climate window. Valkiūnas et al. (2022) described both an increase in prevalence and a range expansion for *P. circumflexum* over the past 40 years (a parasite that primarily infects birds breeding in cavities or closed, dome‐shaped nests). Nesting and roosting within cavities is a key aspect of blue tit behavior (Mainwaring 2011), and we think this might play a role in *Plasmodium* transmission. This hypothesis is strengthened by historic human malaria in Scandinavia being an indoor disease where vectors of *P. vivax* were known to overwinter in homes and transmit the parasite (Huldén et al. 2005). Like *P. vivax* vectors living in Scandinavian homes, vectors harboring *P. circumflexum* could potentially remain in “closed nests” as they potentially provide more consistent and favorable environments in contrast to the less predictable outside environment (Valkiūnas et al. 2022). If *P. circumflexum* vectors are overwintering in these cavities and remaining semi‐active year‐round, then transmission may be possible during a relatively broad climate window, and this window may be expanding due to warmer winter temperatures.

In this study, we have identified an effect of climate warming on the transmission and prevalence of malaria parasites in a population of blue tits. Similar impacts of climate change on vector‐transmitted parasites might be occurring more broadly. For example, theoretical approaches have projected overall significant increases in vector‐transmitted disease risk for humans with ongoing climate change (Colón‐González et al. 2021; Martens et al. 1995; Ryan et al. 2021). Similarly, a meta‐analysis by Garamszegi (2011) showed a strong correlation between temperature anomalies and the prevalence of avian *Plasmodium* across a broad range of bird species and geographic regions. Based on what we could infer from the literature cited by Garamszegi (2011), the studies included in this meta‐analysis mostly lacked long‐term data for each host population. However, the findings parallel what we have observed in Revinge blue tits, and it is important to realize that an overall rise in wildlife malaria prevalence, driven by climate warming, has already happened in our study population.

The effects of a rise in vector‐mediated parasite transmission within a host population will depend on the extent to which the parasites are causing disease, and relatedly, the magnitude of host genetic variation for resistance and/or tolerance (Råberg et al. 2009). Variation in these traits will determine the rate of evolution for increased resistance/tolerance in the face of increased parasite prevalence (Roy and Kirchner 2000). Understanding the factors that underlie both parasite transmission and host response to infection will shed light on wildlife population resilience versus collapse given the ongoing rise of prevalence. As many vectors have highly similar ecologies, and the vectors responsible for transmitting wildlife parasites also might transmit disease‐causing parasites to both livestock and humans (Rocklöv and Dubrow 2020), further unravelling of their dynamics in relation to climate warming will be of utmost importance during the coming years.

## Author Contributions

**Angela Nicole Theodosopoulos:** conceptualization, data curation, formal analysis, investigation, methodology, software, visualization, writing – original draft, writing – review and editing. **Fredrik Andreasson:** conceptualization, formal analysis, investigation, methodology, software, visualization, writing – review and editing. **Jane Jönsson:** data curation, methodology, writing – review and editing. **Johan Nilsson:** investigation, writing – review and editing. **Andreas Nord:** funding acquisition, investigation, writing – review and editing. **Lars Råberg:** investigation, writing – review and editing. **Martin Stjernman:** investigation, validation, writing – review and editing. **Ana Sofía Torres Lara:** data curation, investigation, writing – review and editing. **Jan‐Åke Nilsson:** conceptualization, data curation, funding acquisition, investigation, methodology, project administration, supervision, writing – review and editing. **Olof Hellgren:** conceptualization, data curation, formal analysis, funding acquisition, investigation, methodology, project administration, software, supervision, visualization, writing – review and editing.

## Conflicts of Interest

The authors declare no conflicts of interest.

## Supporting information


**Data S1:** Supporting Information.

## Data Availability

The data that support the findings of this study are openly available in Dryad at https://doi.org/10.5061/dryad.37pvmcvz2.
