# Supplementary material for: Climate‐Driven Increase in Transmission of a Wildlife Malaria Parasite Over the Last Quarter Century
Source: Glob Chang Biol. 2025 Oct 20;31(10):e70550. doi: 10.1111/gcb.70550 (PMC12536333; doi:10.1111/gcb.70550)
Supplement: Supplementary file 1 — Data S1: Supporting Information. [file GCB-31-e70550-s001.pdf]

Climate-driven increase in transmission of a wildlife malaria parasite over the last quarter century

Running Title: Climate change drives parasite transmission

Angela Nicole Theodosopoulos,[1] Fredrik Andreasson,[1] Jane Jönsson,[1] Johan Nilsson,[1] Andreas Nord,[1] Lars Råberg,[1] Martin Stjernman,[1] Ana Sofía Torres Lara,[1,2] Jan-Åke Nilsson,[1] Olof Hellgren[1]

Extended Methods

DNA extraction

From every bird sampled, 20-60µL of blood was preserved in 500µL of SET buffer (0.15M NaCl, 0.05M Tris, and 0.001M EDTA at pH ~8.0). For DNA extraction, we combined ~125µL of the SET buffer/blood mix with 2.5 µL of Thermo Fisher Scientific proteinase K (~20 mg/ml), and 3.5µL of 20% SDS. Samples were then shaken and digested at 56°C overnight in a water bath. We then added 125µL of 4M NH_4Ac to each sample, vortexed, and left samples at room temperature for 60 minutes where they were shaken and spun down every 15 minutes. Afterwards, we spun samples at 13000 rpms for 15 minutes to pellet all precipitate and remove the supernatant to a fresh tube. We then added 500µL of ice cold 95% EtOH to each sample, mixed thoroughly by shaking, then spun samples at 13000 rpms for 15 minutes. Following this procedure, we removed the supernatant and added an additional 250µL of ice cold 70% EtOH to the pelleted sample, then immediately removed the liquid. Samples were dried overnight then dissolved in 20µL of 1xTE buffer. We then quantified samples using a nanodrop, and diluted samples to 25ng/µL for downstream PCR methods.

Multiplex PCR

We used multiplex PCR methods to screen for the presence or absence of infection with each of the three malaria parasite genera (Ciloglu et al., 2019). First, we combined 5µL of Qiagen Multiplex Mastermix™, with 1.8µL of ddH_2O and 2µL of 25ng/µL DNA template, with 0.2µL of each primer as described in

previous literature (Ciloglu et al., 2019). We ran the reaction using the following thermocycler profile: a single cycle at 95°C for 15 minutes, 35 cycles of 94°C (30 seconds), 59°C (90 seconds), and 72°C (30 seconds), followed by a single cycle of 72°C ran for 10 minutes. We used gel electrophoresis to identify infections with each genus based on the expected length of DNA fragments where 2.5μL of the final PCR product mixed with 2μL of stopmix (0.01M EDTA, 15% Ficoll, 0.25% bromophenol, and 0.25% xylene cyanol FF) was ran in a 2% 96-well agarose gel combined with 15μL of Biotium GelRed solution. Gels were run at 80V for a maximum of 55 minutes. All multiplex PCRs were carried out in 96-well plates, with positive controls for each of the three genera, and negative controls. Infection scoring was based on both screens. Low intensity infections can have a "blinking on and off" effect with PCR screens (Hellgren et al., 2004). We therefore scored birds as infected if at least one screening revealed an infection. For samples that did not reveal clear results during their screening (i.e., bands appeared too faint to confidently score) they were scored as "NA."

Nested PCR

We first combined 1.0μL of 25ng/μL of DNA template with the following: 15.4μL of ddH_2O, 1.5μL of $MgCl_2$, 2.5μL of 10x reaction buffer, 2.5μL of 10x dNTP mix, 1μL of each 10 μM primer, and 0.1μL of Taq polymerase. Initial PCRs were run using the following thermocycler profile: a single cycle at 94°C for two minutes, 22 cycles at 94°C (30 seconds), 50°C (30 seconds), and 72°C (45 seconds), and ending with a single cycle at 72°C for 10 minutes. The second PCR was then conducted using 2μL of initial PCR product combined with the same concentrations of reagents as above (except we reduced the quantity of ddH_2O to 14.4 μL). Additionally, we used the nested set of primers and ran the reaction for 35 cycles in the thermocycler.

Cytb sequencing

Amplified PCR products from nested PCRs were prepared for sequencing using an ABI 3100 Sanger Sequencer in the following two steps: (1) Precipitation of PCR products and (2) BigDye™ sequencing preparation. To initially precipitate PCR products we combined the 25μL of PCR product with 11μL of 8M NH_4Ac and 37.5μL of 95% room temperature EtOH. Samples were then shaken and rested at room

temperature for 15 minutes. We then pelleted samples by spinning at 3300rpms at 4°C for 30 minutes. After removing the supernatant, we added 50μL of ice cold 70% EtOH and immediately removed the supernatant followed by spinning the samples upside down at 3000rpms and at 4°C for one minute. We then dissolved the precipitated products in 25μL of ddH_2O.

Sequencing preparation was conducted by combining 2μL of precipitated PCR product with the following: 5.0μL of ddH_2O, 1.5μL of 5x Buffer, 0.5μL of the forward nested PCR primer (10μM), and 1μL of Applied Biosystems BigDye™ Terminator Ready Reaction Mix. We then ran the reaction using the following thermocycler profile: a single cycle at 96°C for one minute, followed by 25 cycles of 96°C (10 seconds), 50°C (5 seconds), and (60°C for four minutes). We then precipitated the final products by combining the 10μL of amplified product with 2.5μL of EDTA (125mM) and 35μL of 95% of room temperature EtOH, shaken, then let rest for 15 minutes. Samples were then pelleted by spinning at 3300rpms and at 4°C for 45 minutes. After removing the supernatant, we added 50μL of 70% ice-cold EtOH then immediately removed the supernatant. We then removed all remaining supernatant by spinning the samples upside-down at 3000rpms and at 4°C for one minute.

References

Ciloglu, A., Ellis, V. A., Bernotienė, R., Valkiūnas, G., & Bensch, S. (2019). A new one-step multiplex PCR assay for simultaneous detection and identification of avian haemosporidian parasites. *Parasitology Research*, *118*(1), 191–201. https://doi.org/10.1007/s00436-018-6153-7

Hellgren, O., Waldenström, J., & Bensch, S. (2004). A new PCR assay for simultaneous studies of *Leucocytozoon*, *Plasmodium*, and *Haemoproteus* from avian blood. *Journal of Parasitology*, *90*(4), 797–802. https://doi.org/10.1645/GE-184R1

Stjernman, M. (2004). *Causes and consequences of blood parasite infections in birds*. PhD Thesis, Lund University, Sweden

Supplementary Tables

Table S1. Blood smears were previously screened for *Haemoproteus* infections using samples from the same population, including one-year old birds (2cy) to estimate prevalence (Stjernman, 2004). Importantly, these estimates show that PCR-methods are not underestimating prevalence during the early years which would have happened had the DNA samples degraded with time.

Year	2cy	Population	N blood smears screened (2cy)	N blood smears screened (population)
1997	0.35	0.49	37	82
1998	0.35	0.49	91	134
1999	0.53	0.56	90	125
2000	0.63	0.72	107	202

Table S2. *Climwin* models tested for each malaria parasite (*Haemoproteus*, *Plasmodium*, and *Leucocytozoon*) using minimum, mean, and maximum daily temperatures and testing both a linear and quadratic relationship with response variables. ΔAICc with the best performing models are tabulated with their associated climate window opening and closing Julian dates. Following *climwin* documentation, we combined all windows that together make up 95% of the model weights (i.e., the 95% confidence set) with associated median opening and closing Julian dates, and probabilities (p conf set) that confidence set windows were false probabilities.

**Parasite**	**Temperature**	**Response**	**ΔAICc best model**	**Open best model**	**Close best model**	**ΔAICc conf set**	**Open conf. set**	**Close conf. set**	**p conf. set**
Haemoproteus	mean	linear	-136.71	382	324	-132.14	382	328	
Haemoproteus	min	linear	-96.50	422	398	-72.52	422	355	
Haemoproteus	max	linear	-139.42	380	333	-139.12	380	334	0.004
Haemoproteus	mean	quadratic	-134.61	382	332	-134.61	382	332	
Haemoproteus	min	quadratic	-115.17	209	199	-115.17	209	199	
Haemoproteus	max	quadratic	-141.84	371	336	-136.76	378	335	
Plasmodium	mean	linear	-90.79	340	330	-88.72	340.5	330	
Plasmodium	min	linear	-65.37	340	328	-58.47	267	208	
Plasmodium	max	linear	-87.32	340	330	-79.71	342	330.5	
Plasmodium	mean	quadratic	-111.47	258	208	-111.33	259	208	
Plasmodium	min	quadratic	-122.60	426	394	-56.55	245	222	
Plasmodium	max	quadratic	-119.02	257	209	-117.77	258	207	0.269
Leucocytozoon	mean	linear	-57.39	420	394	-52.99	438	388	0.138
Leucocytozoon	min	linear	-49.93	435	383	-43.57	444	369	
Leucocytozoon	max	linear	-53.70	435	395	-50.45	437	391	
Leucocytozoon	mean	quadratic	-56.11	420	394	-49.29	437	387	
Leucocytozoon	min	quadratic	-58.13	46	4	-46.39	436	381	
Leucocytozoon	max	quadratic	-52.04	419	395	-44.31	442	387	

Table S3: Number of samples from the study that were typed for *Haemoproteus*, *Plasmodium,* and *Leucocytozoon*, using nested PCR methods, and the total number of unique lineages. Some lineages could not be typed, and this may be due to them being coinfections. All typed samples were also screened using multiplex PCR methods.

**Genus**	**Samples Typed**	**# Lineages**	**Unknown**
Haemoproteus	564	1	0
Plasmodium	94	3	8
Leucocytozoon	83	8	27

Table S4. Tabulation of *Haemoproteus* (HAEM), *Plasmodium* (PLAS), and *Leucocytozoon* (LEUCO) prevalence for one-year-old (2cy) birds and the total population for each year of sampling.

Year	HAEM_2cy	PLAS_2cy	LEUCO_2cy	HAEM_Population	PLAS_Population	LEUCO_Population
1996	0.41	0.93	0.76	0.47	0.95	0.76
1997	0.45	0.41	0.86	0.51	0.65	0.87
1998	0.49	0.28	0.59	0.58	0.38	0.59
1999	0.64	0.68	0.91	0.63	0.66	0.91
2000	0.66	0.56	0.76	0.71	0.60	0.78
2007	0.81	0.67	0.61	0.83	0.72	0.65
2008	0.85	0.35	0.90	0.87	0.46	0.85
2009	0.88	0.57	0.76	0.92	0.65	0.80
2010	0.70	0.66	0.87	0.77	0.68	0.88
2011	0.80	0.53	0.70	0.87	0.67	0.77
2017	0.95	0.86	0.80	0.96	0.90	0.80
2018	0.92	0.84	0.85	0.94	0.86	0.86
2019	0.96	0.79	0.85	0.96	0.86	0.83
2020	0.86	0.80	0.92	0.89	0.84	0.90
2021	0.86	0.81	0.96	0.92	0.89	0.91
